# ImmunTraCkeR^®^ as a reliable TCR repertoire profiling tool to understand immune response and to explore immunotherapy biomarkers

**DOI:** 10.1186/2051-1426-1-S1-P112

**Published:** 2013-11-07

**Authors:** Isabelle Tanneau, Audrey Nondé, Anaïs Courtier, Gilles Parmentier, Marlène Noël, Audrey Grivès, Solène Perez, Nadia Plantier, Jean-François Mouret, Orchidée Filipe-Santos, Manuarii Manuel

**Affiliations:** 1 ImmunID Technologies, Grenoble, France

## 

Over the last decade, the host immunity has emerged as a critical determinant of cancer development and of response to therapy. Great progress has been made in the development of immunotherapies, which leverage the patient’s immune system to reach better clinical outcomes. Immune checkpoint inhibitors, such as anti-CTLA-4 or anti-PD-1/PD-L1, show deep and durable tumor responses in various indications. However, response rates vary a lot depending on the tumor type and remain relatively low. Scientists and clinicians are currently struggling in the research of reliable biomarkers to predict patient response. Studying the development and selection of lymphocyte repertoires is essential to understand the function of the immune system in healthy individuals and in cancer patients. We propose an innovative test called ImmunTraCkeR^®^ to study T cell immune repertoire diversity, based on the detection of V-J rearrangements of the T cell receptor (TCR). Our immune profiling platform allows to monitor immune diversity in blood or tumors and to assess variations between individuals. Past studies have shown the clinical utility of T cell diversity and patient’s immune status in several indications, including metastatic cancers and infections. Here, we performed a study on healthy subjects to evaluate the performances of our immune profiling platform and the stability of T cell repertoire over time. TCR diversity was assessed through the human ImmunTraCkeR^®^ test on genomic DNA extracted from peripheral blood mononuclear cells, obtained from 31 healthy donors. For 4 samples, ImmunTraCkeR^®^ was replicated at least twice to evaluate platform performances. In addition, day-0 and day-21 samples from 27 healthy subjects were tested in blind to further assess ImmunTraCkeR^®^ performances and to evaluate the kinetics of TCR diversity. Unsupervised hierarchical clustering with multiscale bootstrap resampling showed the high repeatability of the platform and high stability of TCR repertoire overtime in healthy subjects. Comparison to cancer patients under treatment is currently ongoing. Results confirm that ImmunTraCkeR^®^ can be used as a powerful and reliable tool to monitor kinetics of T cell repertoire during the course of disease and/or treatment. ImmunTraCkeR^®^ may allow clinicians to better understand their patient’s response and to adapt the therapeutic strategy according to the host’s immune status.

